# Supplementary material for: Deep structured learning for variant prioritization in Mendelian diseases
Source: Nat Commun. 2023 Jul 13;14:4167. doi: 10.1038/s41467-023-39306-7 (PMC10345112; doi:10.1038/s41467-023-39306-7)
Supplement: Supplementary file 1 — Supplementary Information [file 41467_2023_39306_MOESM1_ESM.pdf]

Supplementary Information

Deep structured learning for variant prioritization in Mendelian diseases

Matt C. Danzi, Maike F. Dohrn, Sarah Fazal, Danique Beijer, Adriana Rebelo, Vivian Cintra, and Stephan Züchner

Supplementary Figures

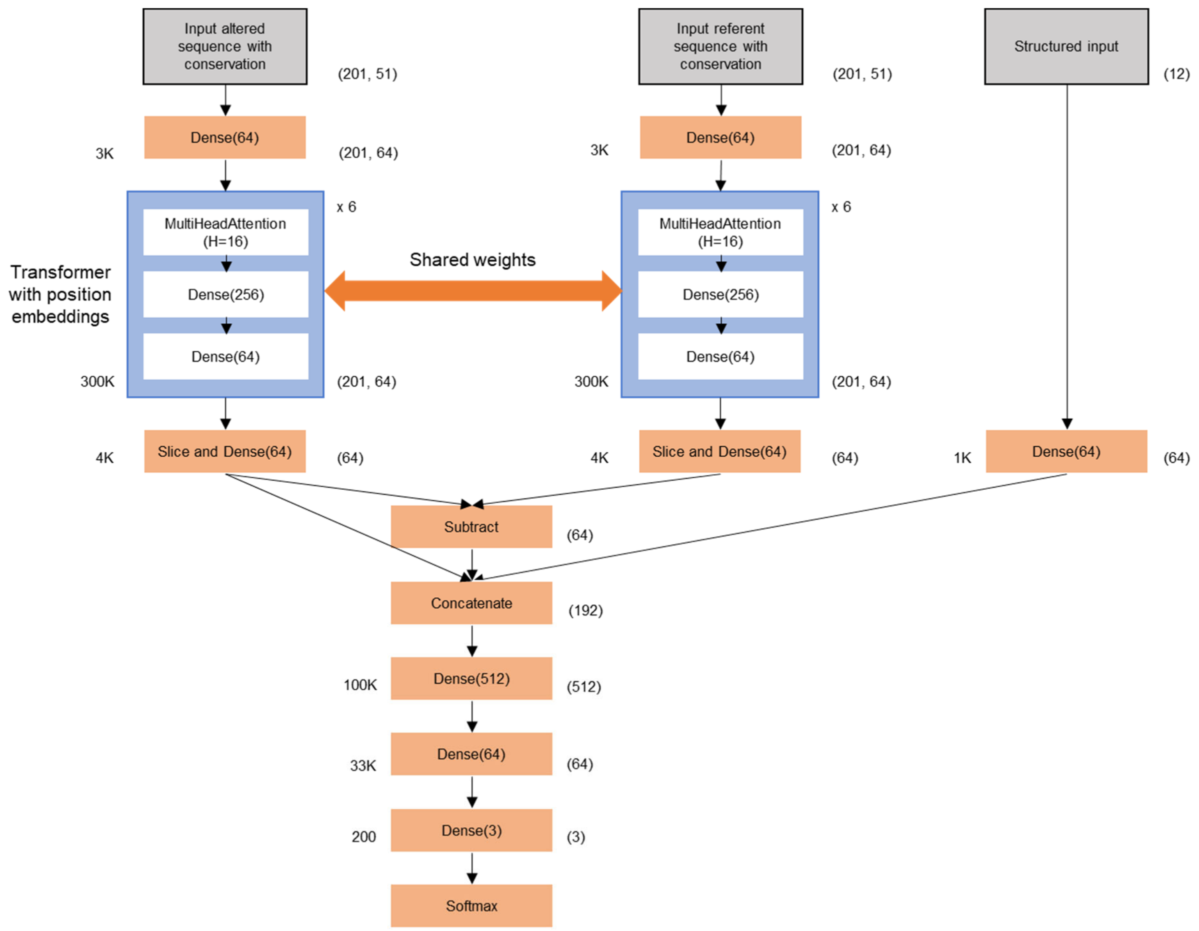

Supplementary Figure 1: MAVERICK sub-model architecture 1. Inputs are shown as grey boxes. Transformer-based layers are shown in blue. Densely-connected linear layers are shown in orange. The number of parameters is shown to the left of each layer. The size of the output of each layer is shown on its right side. The size of each densely-connected linear layer is given in parentheses within the layer. For the multi-head attention layers, sixteen attention heads were used. The weights are shared between the two stacks of transformer layers.

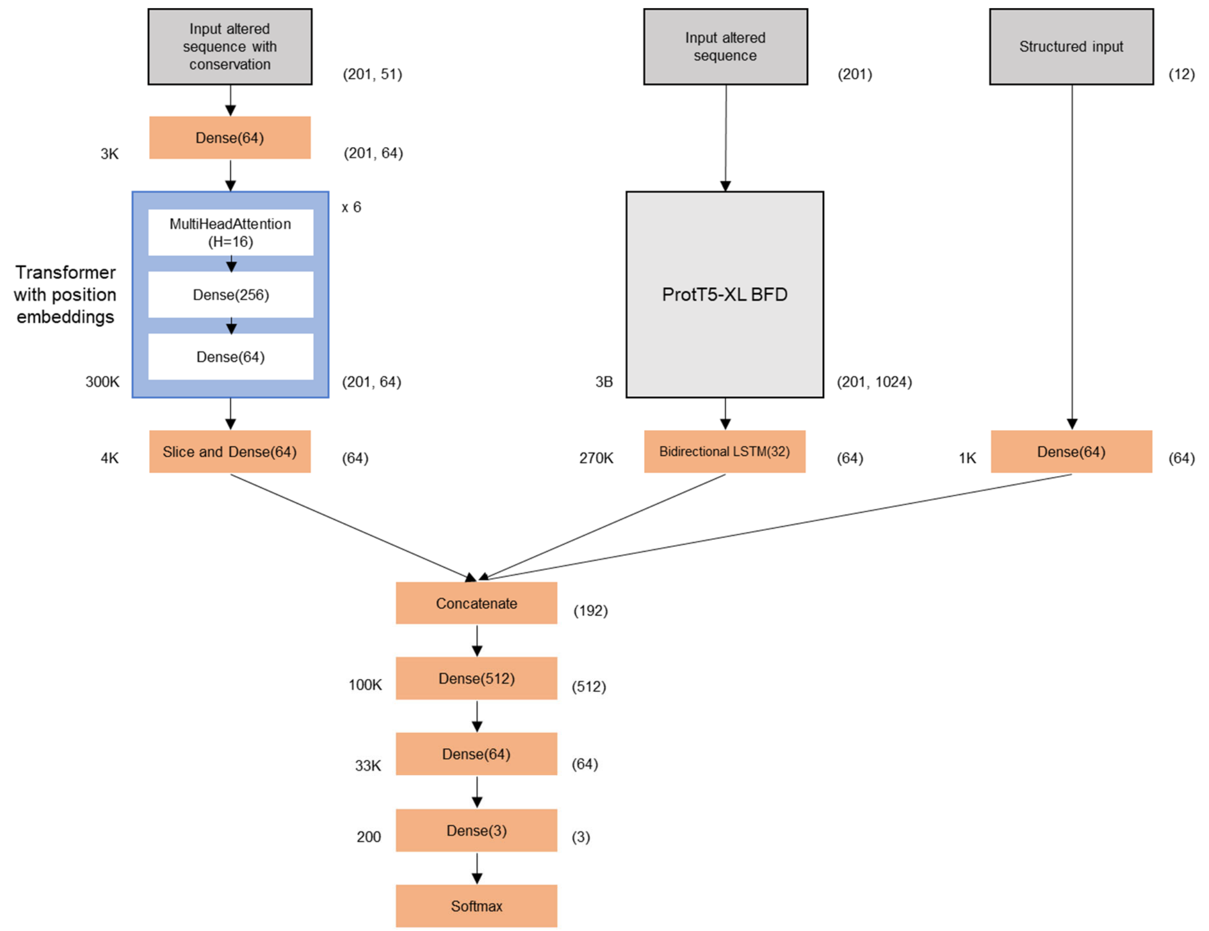

Supplementary Figure 2: MAVERICK sub-model architecture 2. Inputs are shown as grey boxes. Transformer-based layers are shown in blue. Densely-connected linear layers are shown in orange. The number of parameters is shown to the left of each layer. The size of the output of each layer is shown on its right side. The size of each densely-connected linear layer is given in parentheses within the layer. For the multi-head attention layers, sixteen attention heads were used. ProtT5-XL BFD was used as an additional feature extractor in this architecture.

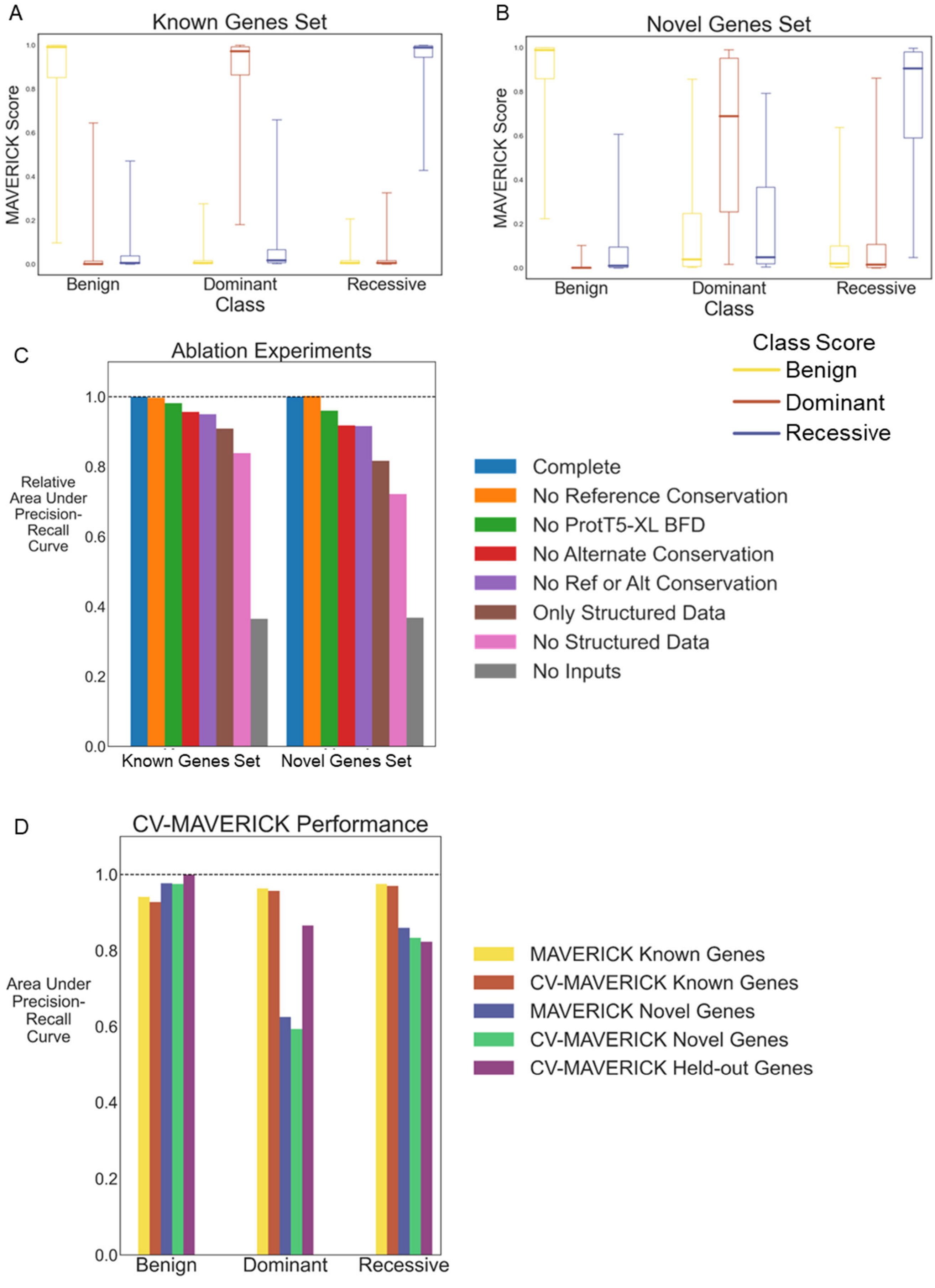

A
Known Genes Set
MAVERICK Score
Benign
Dominant
Recessive
Class
B
Novel Genes Set
MAVERICK Score
Benign
Dominant
Recessive
Class
Class Score
Benign
Dominant
Recessive
C
Ablation Experiments
Relative Area Under Precision-Recall Curve
Known Genes Set
Novel Genes Set
Complete
No Reference Conservation
No ProtT5-XL BFD
No Alternate Conservation
No Ref or Alt Conservation
Only Structured Data
No Structured Data
No Inputs
D
CV-MAVERICK Performance
Area Under Precision-Recall Curve
Benign
Dominant
Recessive
MAVERICK Known Genes
CV-MAVERICK Known Genes
MAVERICK Novel Genes
CV-MAVERICK Novel Genes
CV-MAVERICK Held-out Genes

Supplementary Figure 3: MAVERICK classifies benign, dominant, and recessive variants. A-B) Box plots of distributions of MAVERICK predictions for each of the three classes, separated by true class label. A) Known genes test set. B) Novel genes test set. Box plot elements in show the median as the center line, the 25th and 75th percentiles as limits of the boxes, and the 5th and 95th percentiles as the limits of the whiskers. Outliers are not plotted. C) Relative performance of MAVERICK with different input components ablated by dropout. Performance is measured by the area under the precision-recall curve normalized to the score of the complete MAVERICK model, averaged among the benign, dominant and recessive scores. The ‘Only Structured Data’ condition ablated input from reference allele conservation, alternate allele conservation, and the ProtT5-XL BFD model. The ‘No Inputs’ condition shows random guessing performance. D) Areas under the precision-recall curve for MAVERICK and CV-MAVERICK on the known and novel genes test sets as well as for CV-MAVERICK on its cross-validation held-out genes test set.

A

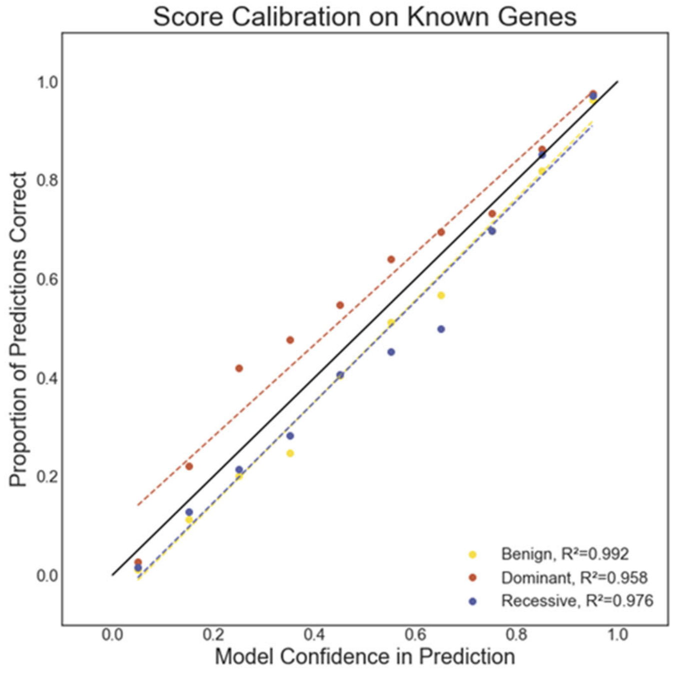

B

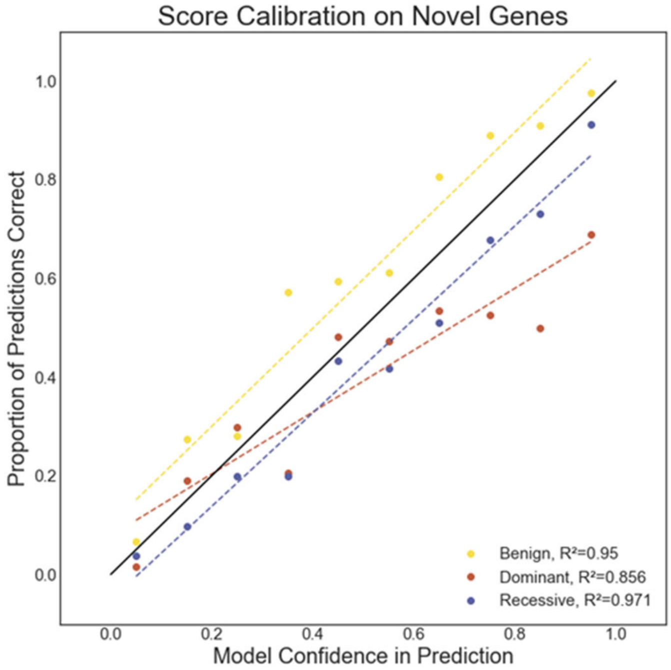

Supplementary Figure 4: MAVERICK's predictions are well-calibrated. A-B) Scatter plots of binned prediction values on the x-axis plotted against the proportion of those predictions for which this was the correct class on the y-axis. A perfectly calibrated model would have all its points fall on the x=y line. Calibration curves above the x=y line indicate under-confidence, while those

under the x=y line indicate over-confidence. A) Calibration curve for the known genes test set. B) Calibration curve for the novel genes test set.

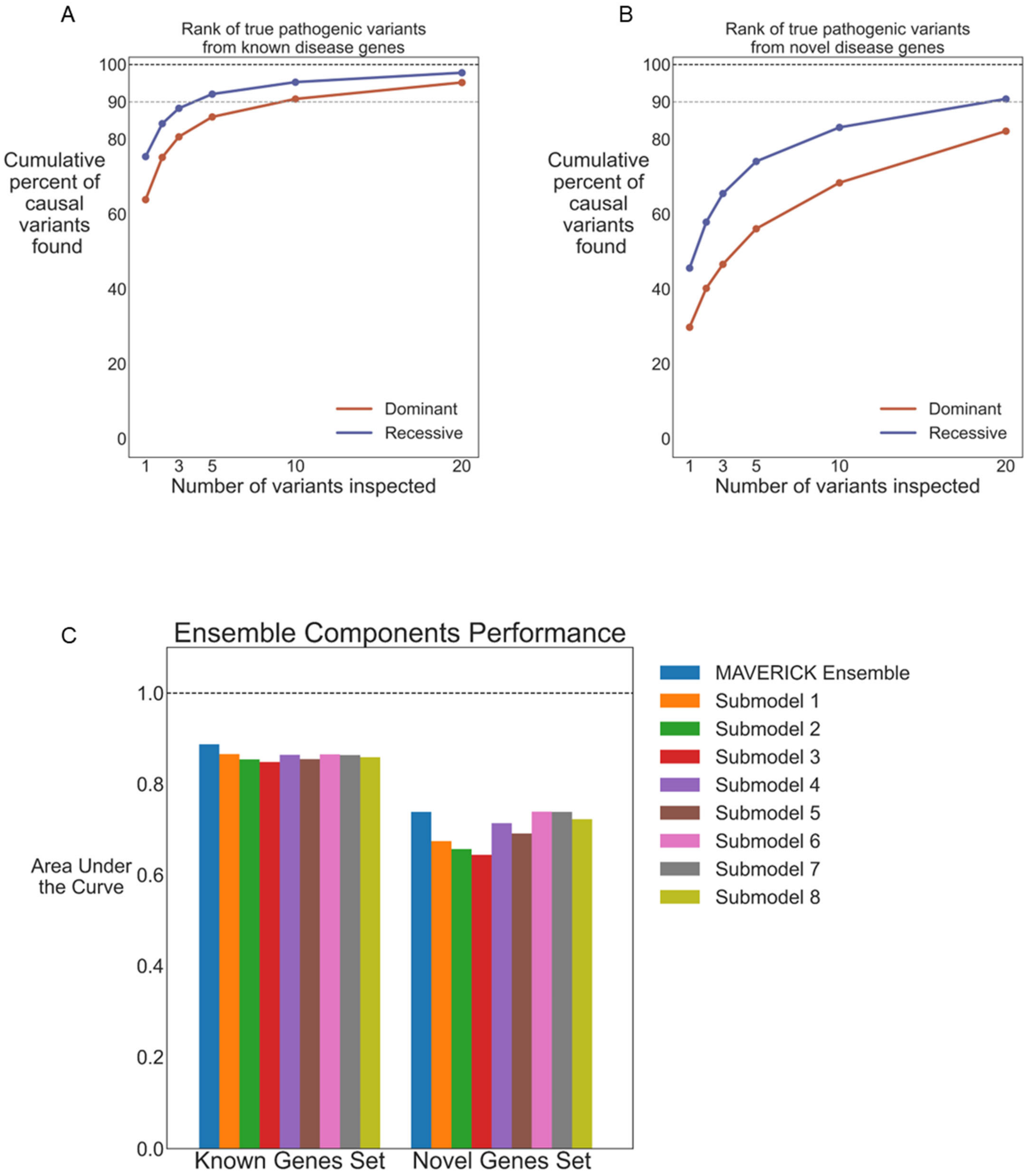

Supplementary Figure 5: MAVERICK reliably prioritizes causal variants. Cumulative proportion of cases solved by MAVERICK's rank ordering of variants when 98 control samples

had pathogenic variants from the known and novel genes test sets spiked in. A) Performance on the known genes test set separated according to the inheritance pattern of the spiked-in variant. B) Performance on the novel genes test set separated according to the inheritance pattern of the spiked-in variant. C) Performance of the individual sub-models that make up MAVERICK's ensemble on this ranking task, quantified by area under the curve.

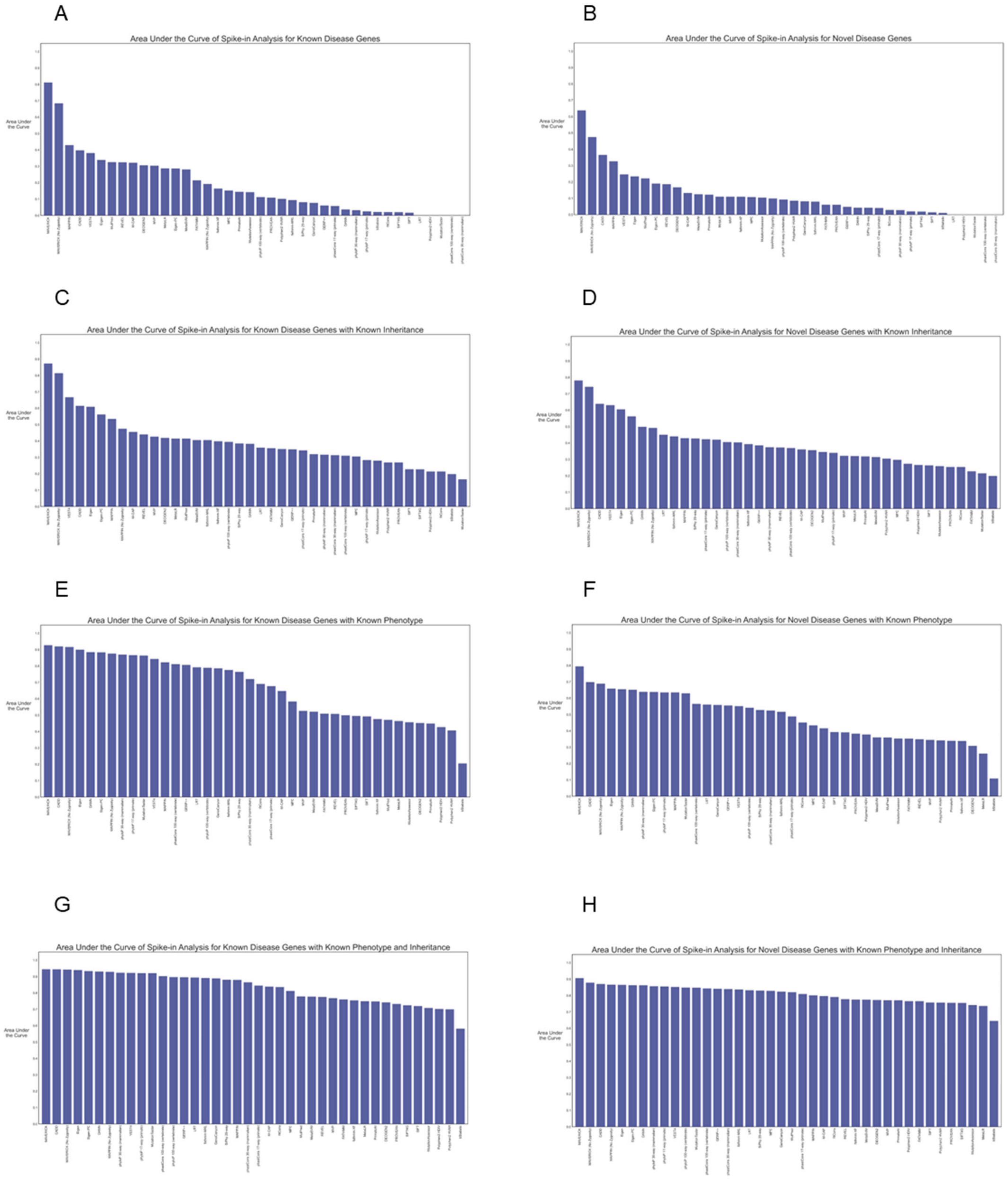

Supplementary Figure 6: MAVERICK outperforms similar tools at variant prioritization. Areas under the curve of cumulative proportion of cases solved by each tool's rank ordering of

variants when 98 control samples had pathogenic variants from the known and novel genes test sets spiked in. This analysis used SNVs only and was restricted to the top 20 ranked variants in each simulated individual. A-B) Performance using only genotype information. C-D) Performance with incorporation of inheritance information. E-F) Performance with incorporation of phenotypic information using GADO. G-H) Performance with incorporation of inheritance and phenotypic information. A, C, E, G) Known genes test set. B, D, F, H) Novel genes test set.

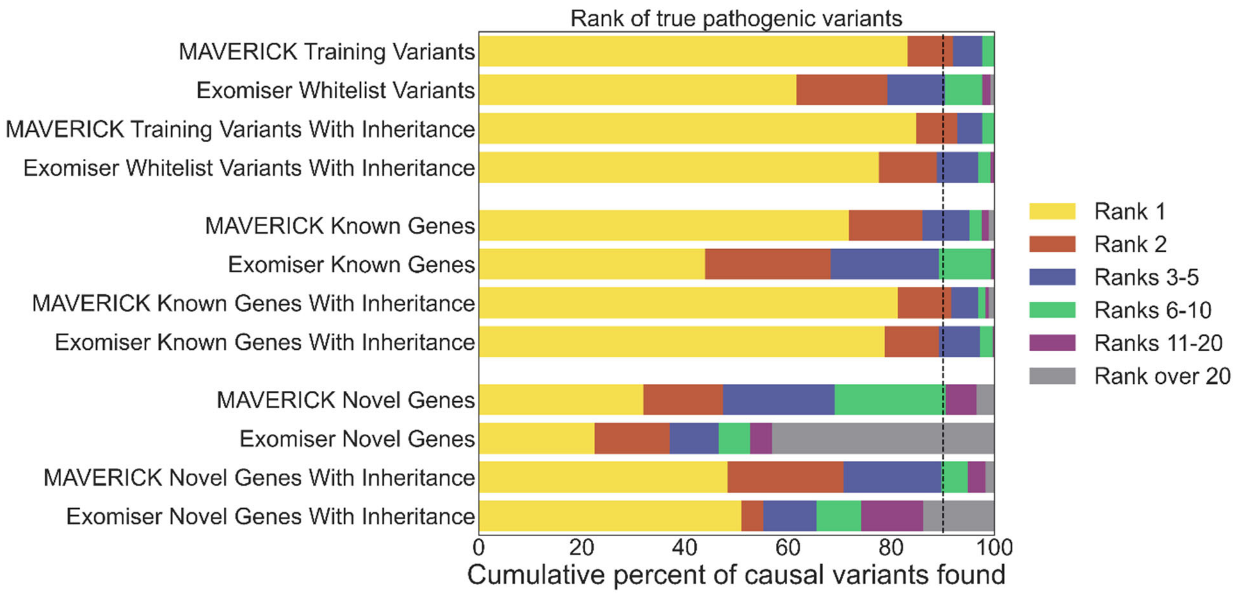

Supplementary Figure 7: MAVERICK and Exomiser reliably prioritize causal variants in real patients. Stacked horizontal bar plot of the cumulative proportion of cases solved by MAVERICK or Exomiser with incorporation of phenotypic information using Phenix. Results are shown with and without incorporation of inheritance information. Top) Results for 125 cases where the causal variant was in both MAVERICK's training set and Exomiser's whitelist of ClinVar variants. Middle) Results for 287 cases where the causal variant was not in MAVERICK's training set or Exomiser's whitelist but lies on a gene which is in MAVERICK's training set and has a known gene-phenotype relationship in Phenix. Bottom) Results for 116 cases where the causal variant lies on a gene neither in MAVERICK's training set nor in Exomiser's whitelist and does not necessarily have a known gene-phenotype relationship in Phenix.

Supplementary Tables

Validation Set

Class Label	Precision	Recall	F1	N	auROC	auPRC
Benign	0.9923	0.9884	0.9903	778	0.9987	0.9996
Dominant	0.9541	0.963	0.9585	108	0.9991	0.9929
Recessive	0.9310	0.9474	0.9391	114	0.9975	0.9789

Known Genes Set

Class Label	Precision	Recall	F1	N	auROC	auPRC
Benign	0.8824	0.8797	0.881	2917	0.9799	0.9411
Dominant	0.9239	0.8902	0.9068	6085	0.9752	0.9632
Recessive	0.9130	0.9431	0.9278	7010	0.9805	0.9751

Novel Genes Set

Class Label	Precision	Recall	F1	N	auROC	auPRC
Benign	0.9343	0.9109	0.9224	1234	0.9623	0.9771
Dominant	0.5930	0.6448	0.6178	183	0.9359	0.6253
Recessive	0.7765	0.7992	0.7877	513	0.9337	0.8598

Supplementary Table 1: Classification performance of MAVERICK on the validation set, the known genes test set and the novel genes test set. For each class in each test set, the number of variants, precision, recall, F1-score, area under the receiver operating characteristic curve, and area under the precision-recall curve are given.

Held-Out Set

Class Label	Precision	Recall	F1	N	auROC	auPRC
Benign	0.9917	0.9818	0.9867	100153	0.997	0.9992
Dominant	0.8948	0.6337	0.7419	13220	0.9802	0.8656
Recessive	0.6797	0.9270	0.7843	13361	0.9788	0.8232

Known Genes Set

Class Label	Precision	Recall	F1	N	auROC	auPRC
Benign	0.8752	0.8536	0.8643	2917	0.9728	0.9272
Dominant	0.907	0.8879	0.8974	6085	0.9705	0.9569
Recessive	0.9055	0.9314	0.9183	7010	0.9766	0.9700

Novel Genes Set

Class Label	Precision	Recall	F1	N	auROC	auPRC
Benign	0.9421	0.8963	0.9186	1234	0.9601	0.9748
Dominant	0.5478	0.6885	0.6102	183	0.9336	0.5935
Recessive	0.7643	0.7836	0.7738	513	0.9200	0.8332

Supplementary Table 2: Classification performance of CV-MAVERICK on the cross-validation held-out genes test set, the known genes test set and the novel genes test set. For each class in each test set, the number of variants, precision, recall, F1-score, area under the receiver operating characteristic curve, and area under the precision-recall curve are given.

**Known Genes Set**	MAVERICK		MAPPIN		
Variant Class	Precision (%)	Recall (%)	Precision (%)	Recall (%)	N
Benign	91.1	89.6	94.1	7.5	2541
Dominant	87.8	86.3	60.5	81.5	2576
Recessive	89.3	91.9	60.1	89.6	2945

**Novel Genes Set**	MAVERICK		MAPPIN		
Variant Class	Precision (%)	Recall (%)	Precision (%)	Recall (%)	N
Benign	94.8	91.0	97.7	8.0	1072
Dominant	62.6	62.1	21.9	59.9	132
Recessive	69.1	79.7	24.1	87.8	286

Supplementary Table 3: Comparison of MAVERICK classification performance to MAPPIN. For each class in the known and novel genes test sets, the number of variants evaluated is given, along with the precision and recall of MAVERICK and MAPPIN.

**Known Genes Set**	MAVERICK		ALoFT		
Variant Class	Precision (%)	Recall (%)	Precision (%)	Recall (%)	N
Benign	50.0	9.1	0.0	0.0	22
Dominant	92.4	86.0	69.4	71.1	1001
Recessive	90.7	95.8	80.1	79.8	1538

**Novel Genes Set**	MAVERICK		ALoFT		
Variant Class	Precision (%)	Recall (%)	Precision (%)	Recall (%)	N
Benign	100.0	22.2	100	11.1	9
Dominant	54.6	64.3	40.0	42.9	28
Recessive	89.3	90.5	75.3	88.5	157

Supplementary Table 4: Comparison of MAVERICK classification performance to ALoFT. For each class in the known and novel genes test sets, the number of variants evaluated is given, along with the precision and recall of MAVERICK and ALoFT.

X-chromosome

Class Label	Precision	Recall	F1	N	auROC	auPRC
Benign	0.9718	0.9727	0.9723	3333	0.9924	0.9955
Dominant	0.3553	0.8801	0.5063	667	0.8849	0.4623
Recessive	0.7383	0.1519	0.2520	1244	0.8749	0.5914

X-chromosome binary

Class Label	Precision	Recall	F1	N	auROC	auPRC
Benign	0.9738	0.9697	0.9717	3333	0.9924	0.9955
Pathogenic	0.9475	0.9545	0.951	1911	0.9924	0.9879

Supplementary Table 5: Classification performance of MAVERICK on the X chromosome. For each class, the number of variants, precision, recall, F1-score, area under the receiver operating characteristic curve, and area under the precision-recall curve are given. These scores are again reported for the "binary" case where dominant and recessive variants are grouped together as "pathogenic".

Feature	Architecture 1	Architecture 2
Trainable parameters	473,539	723,651
Transformer layers	6	6
Transformer output size	64	64
Transformer inner dimension	256	256
Transformer inner activation function	ReLU	ReLU
Transformer output dropout rate	0.1	0.1
Transformer attention dropout rate	0.1	0.1
Number of attention heads	16	16
Maximum learning rate	0.1	0.1
Maximum momentum	0.85	0.85

Supplementary Table 6: Major hyperparameters of the two MAVERICK architectures that were tuned using the validation set.
