# Supplementary material for: Deep structured learning for variant prioritization in Mendelian diseases
Source: Nat Commun. 2023 Jul 13;14:4167. doi: 10.1038/s41467-023-39306-7 (PMC10345112; doi:10.1038/s41467-023-39306-7)
Supplement: Supplementary file 3 — Description of Additional Supplementary Files [file 41467_2023_39306_MOESM3_ESM.pdf]

File Name: Supplementary Data 1

Description: Comparison of MAVERICK to Exomiser in solving real novel cases with and without phenotype information. For each case, the causal gene is shown, along with its mode of inheritance, the patient's HPO terms, and the rank of the causal variant or variant pair when evaluated by MAVERICK using only genotype information, MAVERICK score averaged with HiPhive phenotype score, Exomiser using only genotype information, and Exomiser combined gene-level genotype and phenotype score.

File name: Supplementary Software 1

Description: The publication version of MAVERICK software is provided along with instructions for installation and a demo example. Please see the Readme.md and INSTALL files contained within the zip archive for further information. The actively maintained version of MAVERICK can be found at https://github.com/ZuchnerLab/Maverick.
