# Supplementary material for: Deep structured learning for variant prioritization in Mendelian diseases
Source: Nat Commun. 2023 Jul 13;14:4167. doi: 10.1038/s41467-023-39306-7 (PMC10345112; doi:10.1038/s41467-023-39306-7)
Supplement: Supplementary file 6 — Reporting Summary [file 41467_2023_39306_MOESM6_ESM.pdf]

nature portfolio

Corresponding author(s): Stephan Zuchner

Last updated by author(s): Apr 21, 2023

Reporting Summary

Nature Portfolio wishes to improve the reproducibility of the work that we publish. This form provides structure for consistency and transparency in reporting. For further information on Nature Portfolio policies, see our Editorial Policies and the Editorial Policy Checklist.

Statistics

For all statistical analyses, confirm that the following items are present in the figure legend, table legend, main text, or Methods section.

n/a	Confirmed	
☐	☒	The exact sample size (*n*) for each experimental group/condition, given as a discrete number and unit of measurement
☒	☐	A statement on whether measurements were taken from distinct samples or whether the same sample was measured repeatedly
☐	☒	The statistical test(s) used AND whether they are one- or two-sided<br>*Only common tests should be described solely by name; describe more complex techniques in the Methods section.*
☒	☐	A description of all covariates tested
☒	☐	A description of any assumptions or corrections, such as tests of normality and adjustment for multiple comparisons
☐	☒	A full description of the statistical parameters including central tendency (e.g. means) or other basic estimates (e.g. regression coefficient) AND variation (e.g. standard deviation) or associated estimates of uncertainty (e.g. confidence intervals)
☐	☒	For null hypothesis testing, the test statistic (e.g. *F*, *t*, *r*) with confidence intervals, effect sizes, degrees of freedom and *P* value noted<br>*Give P values as exact values whenever suitable.*
☒	☐	For Bayesian analysis, information on the choice of priors and Markov chain Monte Carlo settings
☒	☐	For hierarchical and complex designs, identification of the appropriate level for tests and full reporting of outcomes
☐	☒	Estimates of effect sizes (e.g. Cohen's *d*, Pearson's *r*), indicating how they were calculated

Our web collection on statistics for biologists contains articles on many of the points above.

Software and code

Policy information about availability of computer code

Data collection

Code to collect the data used to train and evaluate the MAVERICK algorithm is described in the methods subsection "Creation of training, validation, and test sets of variants". The code to replicate this process is provided at our github repository for the manuscript: https://github.com/ZuchnerLab/Maverick.

For completeness, the text from the methods section is reproduced here:
The January 2020 variant summary report was downloaded from ClinVar and used as the primary basis for the training and validation sets. The version of the OMIM database from January 14th, 2020 was downloaded as well. The ClinVar dataset was filtered to identify germline variants with criteria provided and no conflicts in interpretation of pathogenicity (one star or higher). From that set, we selected the following variant types: single nucleotide variants, deletions, duplications, insertions, indels, and microsatellites. We further selected only the variants that were annotated as benign, likely benign, benign/likely benign, pathogenic, likely pathogenic, or pathogenic/likely pathogenic. In order to interpret pathogenic variants as dominant or recessive and select for only Mendelian variants, we further selected only the entries that cited an OMIM phenotype identifier. We used our downloaded version of the OMIM database to map these phenotype identifiers to patterns of inheritance. Most phenotypes in OMIM have only one mode of inheritance (even if the gene has multiple modes of inheritance). Among the cases in which the variant was mapped to a phenotype with both dominant and recessive inheritance annotated, we removed that variant from the set. Additionally, we removed any variants in which the associated OMIM identifier given in ClinVar was deemed to be incorrect. For example, we found entries in which the OMIM identifier pointed to a different gene than the variant was on. We also excluded OMIM terms that were annotated as ‘nondiseases’, ‘susceptibility’, or ‘putative’ (brackets, braces, or question marks). We also included any variants found in gnomAD v2.1.1 in the homozygous state in at least two individuals as benign variants.
To select down to the non-splicing protein-altering variants within this set, we utilized Annovar (version 2018-04-16). We worked with the GRCh37 coordinates for each variant and employed the Gencode V33 Basic annotation of the human genome (lifted over to GRCh37 coordinates). We used Annovar’s annotate_variation.pl and coding_change.pl scripts to identify the protein-sequence changes caused by

each variant on each isoform of each gene they affect. This also provided us with information on variants near splice sites and we chose to remove all variants within 2bp of canonical splice sites. Most protein-altering variants were then seen to affect more than one transcript of the affected gene. We selected a single transcript for each variant as follows: if the variant affects the canonical transcript of the gene (according to gnomAD's definition of canonical transcripts), then use the canonical transcript; if none of the affected transcripts are the canonical one, then use whichever has the highest expression across tissue types in GTEx V7 (using the median of samples as the representative for each tissue type); if multiple genes are affected by this variant, pick the gene whose canonical transcript is affected; if multiple genes have their canonical transcripts affected by this variant, pick the gene whose average expression is highest across tissue types in GTEx V7. In this way, we select down to a single amino acid sequence and how it is altered for each variant.
Next, we collected several numerical annotations for each variant, which are served as structured information to the MAVERICK model. For each variant, we collected the allele frequency and number of times seen as a homozygote among controls in gnomAD v2.1.1; the gnomAD constraint information for the canonical transcript of the gene (regardless of whether the variant affected the canonical transcript or not) in the form of the probability that transcript is loss-of-function intolerant (pLI), probability that transcript falls into distribution of recessive genes (pRec), the probability that transcript falls into distribution of unconstrained genes (pNull), Z-score for missense variants in gene, and Z-score for loss-of-function variants in gene; the pext score from gnomAD; the local constraint score (CCR) for the affected residue; the gene damage index (GDI) score for the associated gene; the RVIS score for the associated gene; and the GERP++ score for the nucleotide harboring the variant. For "residue-level" scores (CCR, pext, and GERP) on indels that span multiple residues, we used the maximum score within the affected span. These sources of structured information were chosen in an effort to supply useful information to the MAVERICK model while minimizing the risk of propagating circularity. As such, these scores were selected because they are based on observations that should be relatively uniform in quality across all genes, regardless of how well-studied a gene is.
The final annotation that we created was the evolutionary conservation track for each gene transcript. This approach was modeled after the procedure used to generate input for NetSurf-P2. For each protein-coding transcript in the Gencode V33 Basic annotation of the GRCh37 genome, MMSeqs2 (Release 11) was used to generate multiple sequence alignments against the August 2018 version of Uniclust90. This was a two-step process, first "mmseqs search" was run with "num-iterations" set to 2 and "max-seqs" set to 2000. Second, "mmseqs results2msa" was run with the default parameters using the output of the first step. These multiple sequence alignments were then run through HHSuite's hhmake utility using default settings except with the parameter "-M" set to "first". Finally, these HHM profiles were parsed into compressed NumPy arrays for easy loading as input to the model.
The protein-altering variants that passed the filtering procedure detailed above were annotated with the appropriate residue-level, transcript-level, and gene-level structured information. The amino acid sequences of the reference and altered protein were saved for each variant as well. This yielded 126,739 variants. One thousand of those variants were randomly selected to serve as the validation set. This contained 778 benign, 108 pathogenic or likely pathogenic dominant, and 114 pathogenic or likely pathogenic recessive variants. The remaining variants composed the training set, which had 99,380 benign, 13,112 pathogenic or likely pathogenic dominant, and 13,247 pathogenic or likely pathogenic recessive variants. The validation set was used for hyperparameter tuning and model selection and as a result, MAVERICK's performance on that set is slightly better than would be expected in general. Therefore, we did not utilize it for primary evaluations.
In order to construct the known and novel genes test sets, we downloaded the January 2021 variant summary report from ClinVar and repeated the above procedure, but at the end removed the 126,739 variants that were already in the training and validation sets. There were 17,942 variants that passed these filters. These could have been newly added to ClinVar, upgraded from zero star to a higher rating, or had an OMIM phenotype term associated with them. The variant set was then split into the 16,012 that fell on genes that had at least one pathogenic variant in the training set (the known genes set) and the 1,930 that fell on genes that had no pathogenic variants in the training set (the novel genes set). The known genes set contained 2,917 benign, 6,085 dominant, and 7,010 recessive variants. The novel genes set contained 1,234 benign, 183 dominant, and 513 recessive variants.
In order to simulate patient phenotypes for each variant in the validation, known genes, and novel genes sets, we exploited the fact that each variant was associated with an OMIM phenotype term due to the manner in which the training and test sets were created. We then used the HPO annotation (downloaded June 21st, 2021) to find the HPO terms associated with each OMIM phenotype. If there were more than five HPO terms associated with any OMIM phenotype, five were randomly selected.
Scoring phenotypes with HiPhive and Phenix was accomplished using the Exomiser REST Prioritiser version 12.1.0. Exomiser data version 2003 (from March of 2020) was used so that the performance of HiPhive and Phenix could be accurately assessed on known and novel genes without the passage of time giving them unfair knowledge of the novel disease genes. The list of up to five HPO terms was passed to the REST prioritiser for each variant, which returns scores between 0 and 1 for every gene. For genes not in Exomiser's annotation set (and therefore without a score), we assigned a phenotype score of 0.5.
Scoring phenotypes with GADO required first converting the set of HPO terms for the OMIM phenotype into the lowest parent term on the HPO graph that was scored by the GADO method. These are referred to as the 'significant' HPO terms. We selected a maximum of five 'significant' HPO terms for each OMIM phenotype. Next, we downloaded the GADO prediction matrix of Z scores from https://molgenis26.gcc.rug.nl/downloads/genenetwork/v2.1/genenetwork_gene_pathway_scores.zip. As described in the GADO paper, known associations between genes and HPO terms were then set to a value of 3. To convert the data from the Z-score range to a more useful range for our purposes, we applied a sigmoid function to compress the scores to a range of 0 to 1. The distribution of values in this matrix was centered on 0.5 and any genes without entries in this matrix were also assigned phenotype scores of 0.5. To compute gene-phenotype scores for sets of 'significant' HPO terms, we took the arithmetic mean of the values of the individual gene-phenotype scores from this matrix.
To combine the phenotype scores from GADO, HiPhive, or Phenix with the MAVERICK score, we took the arithmetic mean of the appropriate MAVERICK score (the 'final score' described above) and the phenotype score for that variant's gene according to each of these tools.
Predictions for all protein-altering SNVs by MAPPIN for hg19 were downloaded from https://doi.org/10.6084/m9.figshare.4639789.
Predictions for all premature stop variants by ALoFT for hg19 were downloaded from https://aloft.gersteinlab.org. Predictions for all missense SNVs by all other tools for hg19 were downloaded from dbNSFP v4.0.

Data analysis

Custom code was written in python to train and evaluate the MAVERICK neural network algorithm. The code to reproduce the training process is provided at our github repository for this manuscript: https://github.com/ZuchnerLab/Maverick.

For manuscripts utilizing custom algorithms or software that are central to the research but not yet described in published literature, software must be made available to editors and reviewers. We strongly encourage code deposition in a community repository (e.g. GitHub). See the Nature Portfolio guidelines for submitting code & software for further information.

Data

Policy information about availability of data

All manuscripts must include a data availability statement. This statement should provide the following information, where applicable:

- Accession codes, unique identifiers, or web links for publicly available datasets
- A description of any restrictions on data availability
- For clinical datasets or third party data, please ensure that the statement adheres to our policy

The MAVERICK program is available under an MIT open source license through the Github repository: https://github.com/ZuchnerLab/Maverick.
The pre-computed scores for all missense and nonsense SNVs in Gencode Basic V33 on GRCh37 and lifted over to GRCh38 have been deposited to Zenodo under the DOI 10.5281/ZENODO.783865953. The training, validation, known genes, and novel genes sets have also been deposited in that repository under the same DOI.
Other datasets and data resources accessed in this work:
OMIM: https://omim.org/
ClinVar: https://www.ncbi.nlm.nih.gov/clinvar/
dbNSFP: https://sites.google.com/site/jpopgen/dbNSFP
gnomAD: https://gnomad.broadinstitute.org/
GTEx: https://gtexportal.org/home/
GERP: http://mendel.stanford.edu/sidowlab/downloads/gerp/index.html
CCR: https://s3.us-east-2.amazonaws.com/ccrs/ccrs/ccrs.autosomes.v2.20180420.bed.gz
ALoFT: https://aloft.gersteinlab.org
MAPPIN: https://doi.org/10.6084/m9.figshare.4639789
Exomiser, HiPhive, and Phenix: https://github.com/exomiser/Exomiser
GADO: https://molgenis26.gcc.rug.nl/downloads/genenetwork/v2.1/genenetwork_gene_pathway_scores.zip
Uniclust90: https://uniclust.mmseqs.com/
HPO: https://hpo.jax.org/app/
GENESIS: https://www.tgp-foundation.org/

Human research participants

Policy information about studies involving human research participants and Sex and Gender in Research.

Reporting on sex and gender	Not applicable to this work.
Population characteristics	Not applicable to this work.
Recruitment	Not applicable to this work.
Ethics oversight	Not applicable to this work.

Note that full information on the approval of the study protocol must also be provided in the manuscript.

Field-specific reporting

Please select the one below that is the best fit for your research. If you are not sure, read the appropriate sections before making your selection.

☒ Life sciences ☐ Behavioural & social sciences ☐ Ecological, evolutionary & environmental sciences

For a reference copy of the document with all sections, see nature.com/documents/nr-reporting-summary-flat.pdf

Life sciences study design

All studies must disclose on these points even when the disclosure is negative.

Sample size	No sample size calculation was performed as no individuals were recruited for this work. We utilized existing cohorts of patients for some analyses, so it is possible that those analyses were under-powered but this is not something we could address. Those patients were collected from the GENESIS database, selecting for individuals previously solved for Mendelian diseases to enable benchmarking efforts. While the number of individuals able to be collected this way is limited, we believe the 644 that we obtained were sufficient for the analyses presented as it is a much greater number than used in the majority of other similar tool development works, where fewer than 100 individuals are typically used.
Data exclusions	To the best of our knowledge, no data were excluded from the analyses in this manuscript.
Replication	Our attempts at reproducibility involved analyzing multiple different cohorts as well as simulated cohorts to ensure that concordant results were obtained. This can be seen in Figure 2 panels A-D,Figure 3 panels B and E, and all panels of Figures 4 and 5. Replicability of the results were demonstrated through the experiments with CV-MAVERICK (Supplementary Figure 3D). Replication of the results presented in Figure 2A-B was performed using the code available on the Github repository and the Google CoLab to ensure that the version of MAVERICK

available to the public performs identically to what is described in the manuscript. All attempts at replication were successful. Beyond these efforts, no further replication was done.

Randomization	In several cases, individuals were segregated into groups non-randomly. This was done to separate individuals based on whether their causal pathogenic variant had been used in the training of MAVERICK or if any variant from that same gene had been used in the training of MAVERICK. In this way, our groupings were not meant to be random subsets, but rather groupings that demonstrated different scenarios that are often encountered.
Blinding	Blinded analyses were not performed in this study. All analyses in this study were objective and quantitative and generally involved the application of simple mathematical equations to the results of classification or ranking tasks, which were automatically applied across each test group. Therefore, there was no need for any blinding during the analysis phase.

Reporting for specific materials, systems and methods

We require information from authors about some types of materials, experimental systems and methods used in many studies. Here, indicate whether each material, system or method listed is relevant to your study. If you are not sure if a list item applies to your research, read the appropriate section before selecting a response.

Materials & experimental systems

n/a	Involved in the study
☒	☐ Antibodies
☒	☐ Eukaryotic cell lines
☒	☐ Palaeontology and archaeology
☒	☐ Animals and other organisms
☒	☐ Clinical data
☒	☐ Dual use research of concern

Methods

n/a	Involved in the study
☒	☐ ChIP-seq
☒	☐ Flow cytometry
☒	☐ MRI-based neuroimaging
